# Ameliorative effect of Silybin on bisphenol A induced oxidative stress, cell proliferation and steroid hormones oxidation in HepG2 cell cultures

**DOI:** 10.1038/s41598-019-40105-8

**Published:** 2019-03-01

**Authors:** Stefania Lama, Daniela Vanacore, Nadia Diano, Carla Nicolucci, Sonia Errico, Marcello Dallio, Alessandro Federico, Carmelina Loguercio, Paola Stiuso

**Affiliations:** 1 Department of Precision Medicine, University of Campania “Luigi Vanvitelli”, Via De Crecchio 7, 80138 Naples, Italy; 2 Department of Experimental Medicine, University of Campania “Luigi Vanvitelli”, Via De Crecchio 7, 80138 Naples, Italy

## Abstract

Bisphenol A (BPA) and silybin are considered xenoestrogens and could interfere with the action of endogenous hormones. It was demonstrated a higher level of BPA in plasma of nonalcoholic steatohepatitis (NASH) patients, compared to those with steatosis (NAFL). We investigated the effect of BPA and silybin, alone or in combination, on proliferation, oxidative stress and steroid metabolism in HepG2 grown in high glucose concentration medium (H-HepG2). Cell viability was assessed by adding 3-(4,5-dimethylthiazol-2-yl)-2,5-diphenyl tetrazolium bromide (MTT). TBARS were quantified by spectrophotometry. The effect of BPA, silybin and their combination on the expression of phosphorilized extracellular signal-regulated kinase (ERK), ERK and Caspase 3 was determined by Western blot analysis. The identifications of lipids and steroid hormones was performed by mass spectrometry. BPA elicited in H-HepG2 oxidative stress and steroid hormones oxidation leading to the formation of metabolite with estrogenic and genotoxic potentials. Silybin ameliorates the harmful BPA-induced effect decreasing glucose uptake and lipid peroxidation. Moreover silybin activates the synthesis of vitamin D3 metabolites and prevent the steroid hormones oxidation. BPA could be considered as an important risk factor in worsening and progression of NAFLD. At the same time silybin could be a valid support to counteract these effects in NASH patients.

## Introduction

Hormones including estrogens, androgens, glucocorticoids, insulin and thyroid hormones (among others) control the tissues and organs functioning in the control of weight and metabolism. Numerous environmental contaminants and commercial products called “Endocrine disrupting chemicals” (EDCs) could mimic or interfere with functions of endogenous hormones. This interference can change or block hormone actions, causing adverse developmental, reproductive, neurological, cardiovascular, metabolic and immune effects in humans. EDCs exert their actions through nuclear hormone receptors, including estrogen receptors (ERs), androgen receptors (ARs), progesterone receptors, thyroid receptors (TRs), and retinoid receptors, among others^1–3^.

BPA is a component of polycarbonate plastics often used in food and beverage containers as well as numerous other products^4^. Several studies have shown that BPA can be release by polycarbonate plastics containers in contact with foods and beverages. The result is the recurring ingestion of BPA and a consequently chronic human exposure^5^. Therefore *in vivo* data confirmed the ability of BPA to bio-accumulate especially in adipose tissue and liver^6^. Trough the increase of oxidative stress, BPA induces inflammation in liver cells resulting in a development and progression of several liver diseases such as non-alcoholic fatty liver disease (NAFLD)^7^. *In vitro* studies also indicated that BPA increased insulin resistance and inflammation in HepG2 cells confirming a direct effect of BPA on liver and adipose tissue omeostasis^8,9^. Recent studies validated that lipid accumulation, induced by BPA exposure, may lead to severe liver pathologies such as nonalcoholic steatohepatitis (NASH) that can evolve to cirrhosis and hepatocellular carcinoma also due to the worsening effect of BPA on this disease. It is known that triglyceride accumulation constitutes the first “hit” needed for the development of this disease. Specially, excessive *de novo* fatty acid synthesis induces the formation of lipotoxic lipid intermediates that contribute to the pathogenesis of NAFLD. Recently we demonstrated a higher increase of Bisphenol A in plasma of NASH patients, compared to those with sample steatosis (NAFL)^10^. Moreover there was a statistically significant association between Bisphenol A plasma levels and the histological picture of inflammation according to the evaluation of lobular inflammation and ballooning^10^.

Various specialized tissues can use cholesterol as the building component of the synthesis of steroid hormones, oxysterols, or bile acids. The steroid hormones *de novo* synthesis starts when the cholesterol is transported by translocator protein of 18 kDa (TSPO) and steroidogenic acute regulatory protein (STAR) into the inner mitochondrial membrane and is converted to pregnenolone by CYP11A1 (cholesterol side-chain cleavage)^11^. Then pregnenolone is converted to progesterone by 3β-hydroxysteroid dehydrogenase either in the mitochondrion or the smooth endoplasmic reticulum. Steroid hormone synthesis is controlled by the activity of several highly substrate-selective cytochrome P450 enzymes and a number of steroid dehydrogenases and reductases. The link of estradiol to ERα activates the steroid biosynthesis inducing the phosphorylation of STAR and TSPO and facilitating the import of cholesterol into the mitochondria.

Extract from the seeds of milk thistle [Silybum marianum] is a widely used traditional herbal/dietary supplement for its strong anti-hepatotoxic activity against almost any kind of human liver damage/toxicity^12,13^. The major biologically active compound of milk thistle is Silybin (Sil), a polyphenolic flavonoid that is safe and well-tolerated^14–16^. It protects the liver from drug or alcohol-related injury^17,18^. In our precedent study we demonstrated that chronic treatment of NASH patients with Sil-based food integrator, induces a decrease of serum lipid peroxidation and restoration of a correct serum values of free cholesterol, lysophosphatidylcholine, sphingomyelins, and phosphatidylcholine^19^.

Thanks to the knowledge of the scientific literature demonstrating the ability of BPA to induce oxidative stress and potentially increase the proliferation rate of different cell types we investigated the *in vitro* effects of BPA on proliferation, oxidative stress and impaired synthesis of steroid hormones in HepG2 cell line and the capacity of Sil to prevent the effects caused by BPA.

## Results

### BPA promotes proliferation and oxidation effects in HepG2 cells and Sil counteract its effects

**Figure 1:**
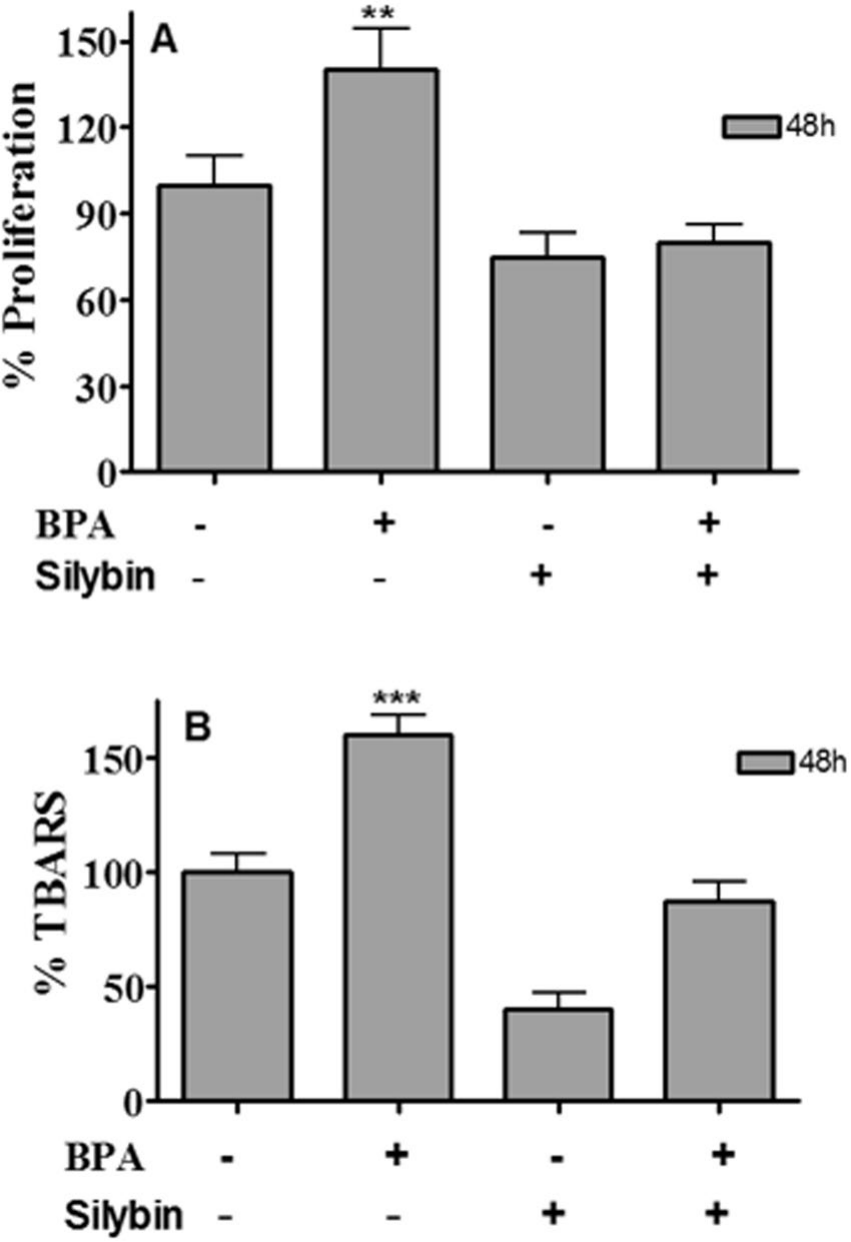
Effects of the treatment with silybin alone or in combination with BPA on growth inhibition of H-HepG2. (**A**) Effect of BPA (0.05 μM), Sil (68 μM) and BPA/Sil combination on cell viability expressed as a percentage of proliferation of the H-Hep-G2. Cell viability was assessed after 48 h by MTT assays. (**B**) Lipid peroxidation evaluated by TBARS after 48 h of treatment of H-Hep-G2 cells with BPA. Abbreviations: BPA: bisphenol A; TBARS: Thiobarbituric Acid-Reactive Species; Sil: silybin.

**Figure 2:**
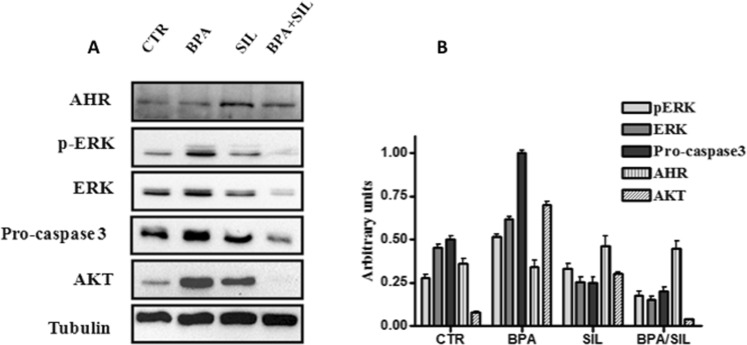
Ahr expression in the BPA, silybin and BPA/silybin treated H-HepG2 cells. (**A**) Evaluation of protein expression mediated by BPA, Sil, alone and in combination with BPA, after 72 h of H-HepG2 treatment. (**B**) Densitometric quantization of the analyzed proteins. Abbreviations: Ahr: aryl hydrocarbon receptor; BPA: bisphenol A; CTR: controls; SIL: silybin; p-ERK: phosphorilized extracellular signal-regulated kinase; ERK: extracellular signal-regulated kinase; AKT: protein kinase B; Sil: silybin.

In a recent manuscript of our group we showed that low concentrations of BPA induced a time and concentration-dependent increase of proliferation and lipid peroxidation only in H-Hep-G2 cells^10^. In particular 0.05 µM BPA, after 48 hours of treatment, induced a significantly increase of proliferation about 2.5 fold (P = 0.0013) compared to untreated cells (data not shown). It is known the antioxidant and cytotoxic capability of Sil, a flavonolignan extracted from milk thistle, in a liver cancer cells^20^. We evaluated the Sil activity on H-HepG2 cells proliferation compared to the effects induced by BPA. The growth inhibition effect induced by Sil (68 μM) on H-HepG2 alone or in combination with BPA (0.05 µM) after 48 hours of treatment is shown in Fig. 1. The results demonstrated that Sil was able to significantly counteract the H-HepG2 BPA-induced cell proliferation. We assayed the lipid peroxidation status of H-HepG2 treated with BPA, Sil and BPA/Sil combination trough the evaluation of toxic reactive aldehydes with thiobarbituric acid reactive substances (TBARS) assay. The BPA induced a significant increase of TBARS concentration (P = 0.0003) of about 1,6-fold compared to control, whereas Sil alone and in combination with BPA reduced of 5 and 2-fold respectively the TBARS value in comparison to BPA treated H-HepG2 cells. Moreover the consumption of glucose evaluated in the medium of Sil and BPA/Sil combination treated cells compared to control cells were decreased of 40 and 25% respectively. Whereas in BPA treated H-HepG2 cells the remaining glucose amount in the growth medium was greater of about 50% compared to control cells. This data showing that Sil is capable to interfere with the uptake of glucose induced by BPA. However, Sil alone or in combination with BPA decreases the protein expression of key molecules involved in the regulation of proliferation and survival: extracellular signal-regulated kinase (ERK), p-ERK and protein kinase B (AKT) (Fig. 2) and induces apoptosis trough the activation of caspase 3. In fact, the expression level of pro-caspase3, a pro-apoptotic protein inactive form, in Sil and Sil /BPA combination treated H-HepG2 cell compared to BPA treated cells decreased.

### BPA and Sil modulate the aryl hydrocarbon receptor activity in HepG2 cells

**Figure 3:**
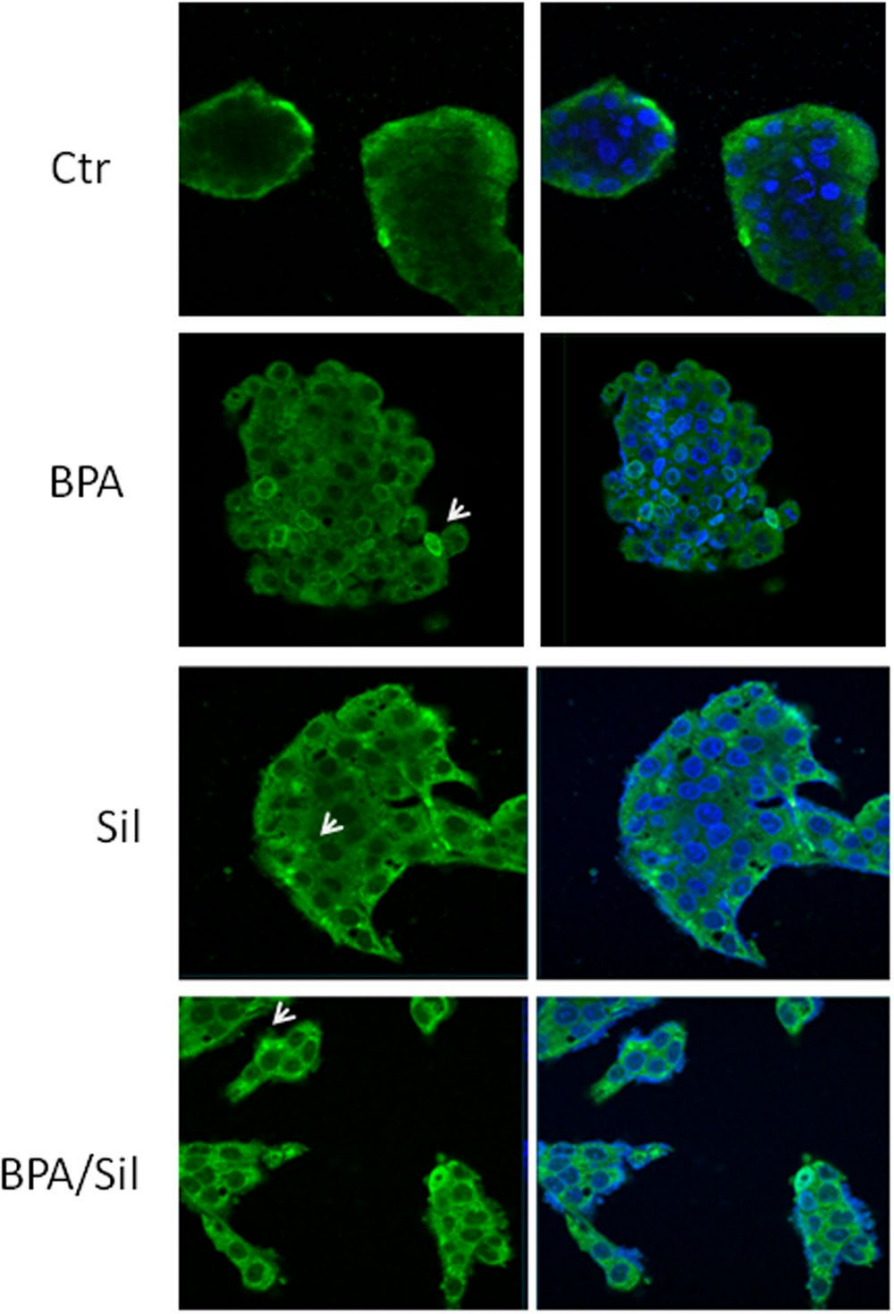
Sub-cellular localization of Ahr. Confocal microscopy pictures of H-HepG2 cells treated without (CTR) and with BPA, Sil and BPA/Sil. The cells were treated for 72 h with BPA 0.05 μM, Sil 68 μM. Green = Ahr and Blue = DAPI. Abbreviations: DAPI: 4′, 6-diamidino-2-phenylindole; CTR: controls; BPA: bisphenol A; Sil: silybin.

The EDCs could influence the ER signaling through stimulation of aryl hydrocarbon receptor (Ahr)^21^. Ahr is a ligand-activated transcription factor that mediates the biologic and toxic effects of its xenobiotic ligands. We examined the expression of Ahr in the BPA, Sil and BPA/ Sil treated H-HepG2 cells (Fig. 2). Sil alone, or in combination with BPA, increases the Ahr expression compared to BPA treated cells. Therefore we evaluated the Ahr sub-cellular localization by confocal microscopy. In Fig. 3 we reported the representative fields of H-HepG2 cells, treated without and with BPA, Sil and BPA/ Sil combination. BPA induced a cytoplasmic-nucleo shuttling of AhR compared to the untreated H-HepG2 (controls). While Ahr to accumulate in the cytoplasm in both Sil and BPA/Sil combination treated H-HepG2 cells, suggesting that Sil, binding the receptor, could blocke its activation.

### BPA induces the steroid hormone de novo synthesis and Sil inhibits BPA-triggered activation pathways

**Figure 4:**
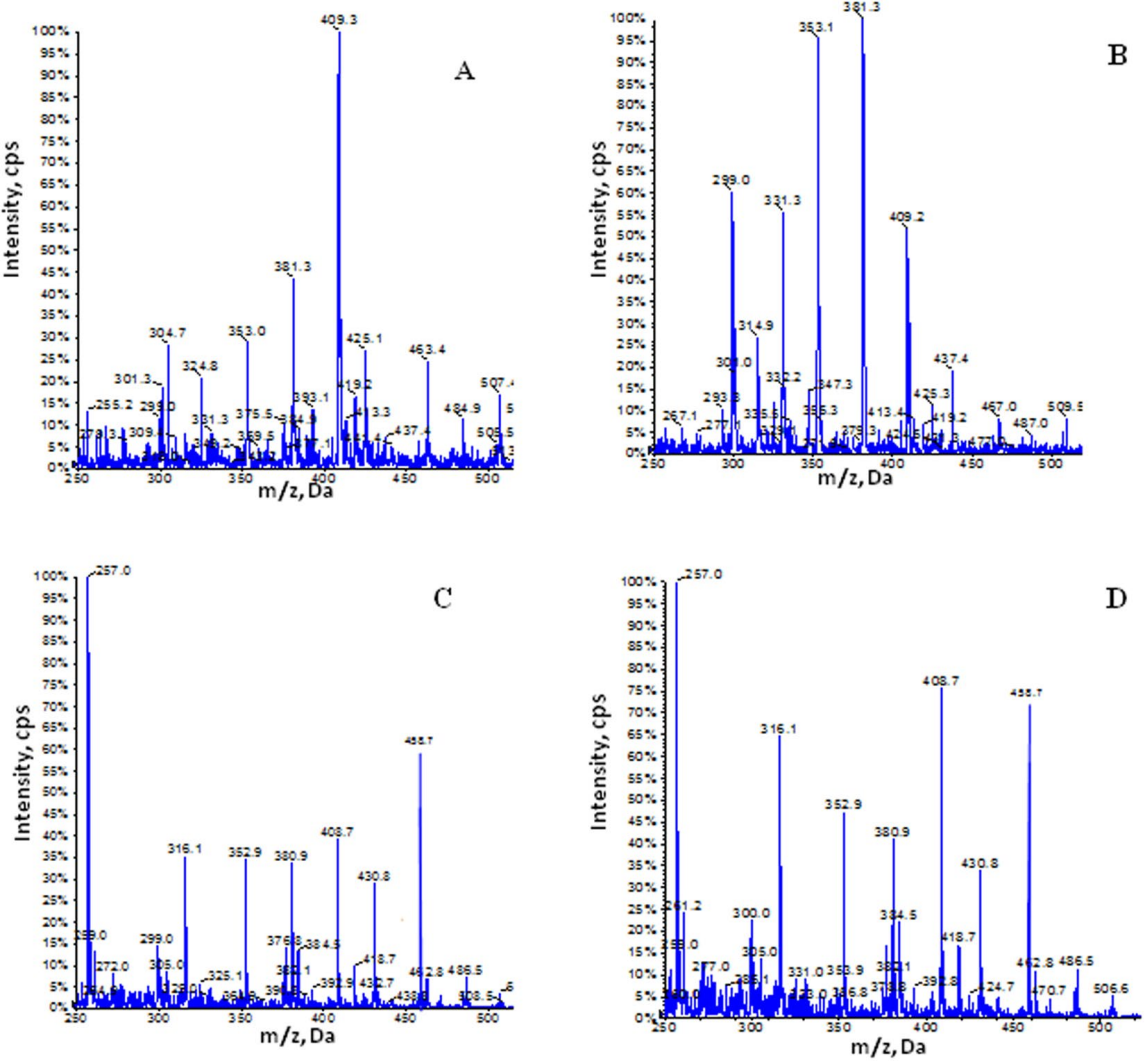
Positive mass spectra of lipid extracted from media of H-HepG2 cells. Positive ion electrospray mass spectra of lipid molecular species in lipid extracts from medium of the H-HepG-2 without (panel A) and with BPA (panel B), Sil (panel C) and BPA/Sil combination (panel D). Aliquots of chloroform extracts were analyzed directly by electrospray as described in method section. Selected peaks are indicated by their *m*/*z* values. For detailed peak assignments see Table 1. Abbreviations: SIL: silybin; BPA: bisphenol A.

**Figure 5:**
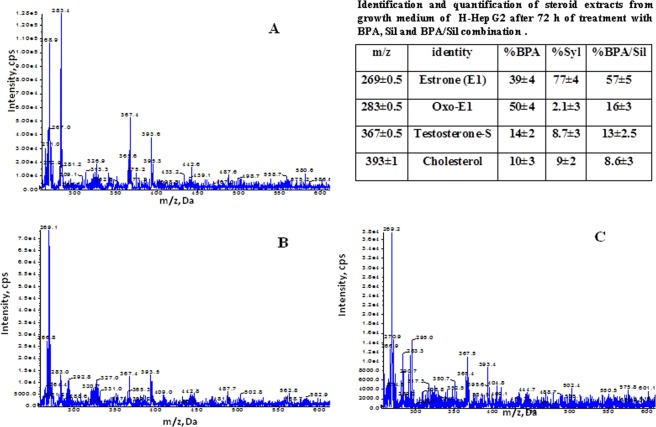
Negative mass spectra of lipid extracted from media of H-HepG2 cells. Negative ion electrospray mass spectra of steroid molecular species extracts from medium of the H-HepG-2 coltured with BPA (panel A), Sil (panel B), BPA/Sil combination (panel C). Aliquots of chloroform extracts were analyzed directly by electrospray as described in method section. Selected peaks are indicated by their *m*/*z* values. For detailed peak assignments see Table 1. Abbreviations: SIL: silybin; BPA: bisphenol A.

**Figure 6:**
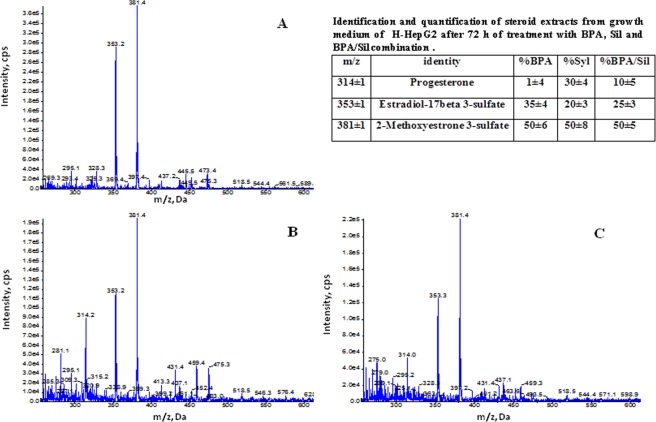
Positive ion mass spectra of steroid molecular species in lipid extracts from media of H-HepG2 cells treated with BPA (**A**), silibin (**B**), BPA/Silibin combination (**C**). Aliquots of by solid phase extraction were analyzed directly by into a triple quadrupole instrument equipped with a TurboIon electrospray source. In the inset were reported the assignments of representative peak. Abbreviations: Sil: silybin; BPA: bisphenol A.

In Fig. 4(A–D) is reported one representative mass spectra of lipid extracted from media of H-HepG2 cells treated without (controls) and with BPA, Sil, and BPA/ Sil combination. In all spectra are evident: 17β-estradiol 3-sulfate (353.1 ± 1 *m/z*), methoxyestrone 3-sulfate (381.3 ± 1 *m/z*), and cholic acid peaks (409.3/408.7 ± 1 *m/z*). Instead the peaks corresponding to progesterone (316.1 ± 1 *m/z*), and 7-dehydrocholesterol (384.3 ± 1 *m/z*) and vitamin D3 derivate compounds (range between m/z 430–486 ± 1) were present only in the media of Sil and BPA/Sil treated H-HepG2. Moreover the peak corresponding to 17-hydroxyprogesterone (*m/z* 331.3 ± 0.2) was clearly evident in the media of BPA treated H-HepG2 cells. In Figs 5 and 6 are reported the negative and positive ion mass spectra of samples obtained by steroid solid phase extraction of media of the H-HepG2 treated with BPA, Sil and BPA/Sil combination. In all negative ion mass spectra (Fig. 5) major representative peaks were: estrone (E_1_ *m/z* 269 ± 1), 16α-oxoestrone (16α-Oxo-E_1_ 283.3 ± 1 *m/z*), testosterone sulfate (TS 367.4 ± 1 *m/z*) and cholesterol (393 ± 1 *m/z*). The percentage of the E_1,_ 16α-Oxo-E_1_ and TS evaluated by peak area of the mass spectra is reported in the inset of Fig. 5. The percentage of 16α-Oxo-E_1_ was significantly decreased in Sil and BPA/Sil treated H-HepG2 cells of 15 and 2-fold respectively compared to the BPA H-HepG2 cells. While in all positive ion mass spectra (Fig. 6) were presented estradiol-17beta 3-sulfate (353 ± 1 *m/z*) and 2-methoxyestrone 3-sulfate (381 ± 1 *m/z*). Only in the positive ion mass spectra of Sil and BPA/Sil was presented the progesterone (314 ± 1 *m/z*). The 17β-E_2_S/2-MeO-E_1_ ratio was 0.8 ± 0.03 in BPA treated cells, whereas was decreased at 0.6 ± 0.025 in Sil and BPA/Sil treated cells.

## Discussion

We evaluated the effect of low concentration of BPA on proliferation, oxidative stress and steroid hormones metabolism and the protective effect of Sil, a natural compound, in HepG2 cell line.

In the study design we decided to evaluate the antioxidant/antiproliferative effects of Sil in comparison to control cell cultures without other comparison with alternative antioxidant compounds such as vitamin E. The reason of this choice was based on the possibility to use Sil as therapeutic approach for liver diseases due to its efficiency and relative low risk of side effects^20^. On the contrary other compounds such as vitamin E are associated to relative inefficiency in the control of liver diseases, in particular NAFLD of adult patients^22^. Moreover there is a lack of well designed randomized controlled trials that proved its efficiency such as to recommend the use of this antioxidant as a promising therapeutic strategy for NAFLD.

It’s known that BPA, in presence of ERα, acts as an E2 (17β-estradiol) mimetic compound inducing cancer cells proliferation^23^. For the experiments we used HepG2 cell line that expresses only ERα^24^. Our data demonstrated that BPA induced cell proliferation *in vitro* only in presence of high glucose concentration. BPA linking the ERα receptor could increase the expression of glucose transporters (GLUT)-4, the glucose uptake and consequently induce fast cell proliferation. Moreover the ERα activation induces proliferation by ERK/mitogen-activated protein kinase (MAPK) and phosphatidylinositol-3 kinase (PI3K)/AKT pathways^25^ and reduces the pro-apoptotic caspase-3 activation. Our results demonstrated that BPA induced an increase the ERK/ERKp and AKT expression and reduced the pro-caspase 3 activation. Despite Sil is considered a xenoestrogen it doesn’t seem to act trough the ERα signaling. In fact it is known the decrease of cellular glucose uptake due to the interaction with GLUT transporters and the inhibition of the constitutive phosphorylation of ERK1/2^26^. We demonstrated that Sil alone or in combination with BPA in our experimental model (H-HepG2) counteracts the BPA proliferation effect both decreasing the glucose uptake and phosphorylation of ERK1/2. Moreover it promotes apoptotic cell death by caspase-3 activation, reducing proliferative effects induced by BPA.

Ahr, activated by a wide variety of hydrophobic ligands, could indirectly regulate the ER signaling^27^. AhR is inactive in a cytosolic complex whit Hsp90 and p23. Similarly, ERs are maintained in a complex that includes heat shock protein (HSP)-90 and p23 in the absence of E_2_^21^. Upon ligand binding, AhR migrates into the nucleus and heterodimerizes with aryl hydrocarbon receptor nuclear translocator (ARNT), and binding the DNA stimulates transcription of target genes^28^. In our experimental condition is interesting to note that Sil alone or in combination with BPA increases the Ahr cytosolic expression evaluated both by Western Blot analysis and confocal microscopy. Than the probably mechanism by which Sil interferes with proliferative effect of BPA could be through the Ahr inactivation. In literature were reported that exposure to low doses of BPA can affect de novo synthesis of steroid hormones by increasing the expression of genes involved in this process^29,30^. The analysis, by mass spectrometry, of media H-HepG2 suggests the presence of molecules belonging to sterols family activated both BPA and Sil. In the Table 1 all metabolites evidenced in the media of the H-HepG2 treated with and without BPA and Sil were reported.

**Table 1:** Assignments of the *m/z* ratios detected in the electrospray mass spectra of the lipid extracts of medium of HepG2 cells treated without and with BPA, Sil, BPA/Sil.

m/z	Identity
316.1 ± 1	C_21_H_31_O_2_ (progesterone)
331.3 ± 1	C_21_H_30_O_3_ (17-hydroxyprogesterone)
353.1 ± 1	C_18_H_24_O_5_S (Estradiol-17beta 3-sulfate)
381.3 ± 1	C_19_H_25_O_6_S (2-Methoxyestrone 3-sulfate)
384.5 ± 1	C_27_H_44_O (7-dehydrocholesterol)
*409.3 ± 0.2/408.7 ± 1	C_24_H_41_O_5_/C_27_H_46_ONa _/_C_27_HO_2_ (cholic acid/cholesterol Na+/27-hydroxy-cholesterol)
430.8 ± 1/458.7 ± 1/463.4 ± 1	1,25-dihydroxyvitamin D3 analog

^*^Identification is not unique (it could be possible plus identities).

BPA: Bisphenol A; Sil: silybin.

In particular the representative peaks, present in all spectra, were the 17β-estradiol 3-sulfate and methoxyestrone 3-sulfate, derived via steroid sulfotransferase activation (EC 2.8.2.15). As sulfated estrogens are unable to bind the estrogen receptors and the sulfate conjugation may protects cells and tissues from an excess of active estrogens, on the other hand 17β-estradiol 3-sulfate could be converted as needed to the more active estradiol. The synthesis of methoxyestrone 3-sulfate in human liver may occur through two subsequently steps: (1) hydroxylation of E_1_-sulphate (2-OHE_1_) catalyzed by cytochrome P (CYP)1A1 or CYP1B1; (2) methylation of 2-OHE_1_-sulphate to methoxy estrogens by catechol-*O*-methyltransferase enzyme. The estrogen quinones have been postulated to be a factor in mammary carcinogenesis, whereas the methylation of catechol estrogens reduces the potential for DNA damage and increases the concentration of an antiproliferative metabolite. Indeed in the media of the BPA treated H-HepG2 cells was evident the 17-hydroxyprogesterone, metabolite derived from pregnenolone by CYP17 and 3β- hydroxysteroid dehydrogenase enzyme (HSD) activity and lead to the final synthesis of estrone or estradiol by CYP19 activity (aromatase). In the media of the Sil treated H-HepG2 cells this metabolite was missing, on the contrary was detected only the progesterone. It was biosynthesized from the pregnenolone by 3β-HSD, and lead to the corticosteroids synthesis. The proliferative effects inducted by BPA are not due only to the ERα interaction, but also to the ex-novo synthesis of active estrogen metabolites. In fact to demonstrate the role of BPA on the estrogen synthesis we performed on culture media a steroid solid phase extraction. The oxidative metabolism of estrone leads to the formation of several metabolites, such as hydroxyestradiol that can undergo metabolic redox cycling to generate free radicals such as superoxide and the reactive semiquinone/quinone intermediates, which cause DNA damage and with an important role in carcinogenesis. These metabolites covalently bind to estrogen receptors, thus stimulating cell proliferation. The analysis of negative ion mass spectrometry of the samples obtained by solid extraction shown the presence of 16-Oxo-estrone (16α-Oxo-E_1,_ 283 ± 1 m/z), an unexpected estrone-oxidate. The percentage of Oxo-estrone increased in the media of BPA treated cells of 14-fold compared to the Sil treated H-HepG2 cell. Interesting in the BPA/Sil combination treated cells, Sil counteracts the Oxo-estrone formation inducted by BPA. Several experimental studies point towards a direct role of vitamin D in modulating liver inflammation and fibrogenesis. In literature were reported the anti-fibrotic, anti-inflammatory and insulin-sensitizing properties exerted by Sil on the chronic liver disease like NAFLD and NASH^20,31^. This properties were probably due to synthesis ex-novo of 1,25(OH)_2_D_3_ founded in the positive ion mass spectra of media Sil treatment. The hidroxy-D3 derivate biosynthesis may be due to increase of liver microsomal enzyme of CYP family.

In the present study we demonstrated that treatment of HepG2 cells with “safe” dose of BPA: (1) increases of proliferation rate only in presence of high glucose concentration; (2) regulate the ERK/MAPK and the PI3K/AKT pathways; (3) induces an increase of oxidative stress and 4) induces an increase of steroid hormones oxidation. These preliminary data suggest that BPA need to be considered as tangible hepatic risk factor. This observation confirms that BPA is an environmental factor able to determine a worsening of NAFLD, especially along with obesity and diabetes. Then positive BPA concentration in human blood may represent a predictive marker of increased risk of of NAFLD progression, particularly in type 2 diabetes mellitus patients. On the other hand the toxic effect elicited by BPA on H-HepG2 cells was reverted by Sil that induced a decrease of: (1) glucose uptake; (2) cell proliferation; (3) lipid peroxidation. Moreover Sil activated the synthesis of vitamin D3 metabolite from cholesterol and prevented the steroid hormones oxidation. For these reasons this molecule could be considered a therapy support for NAFLD and NASH patients.

## Methods

### Cell Culture preparation

Human hepatocellular carcinoma cells (HepG2, HB-8065) were obtained from the American Type Culture Collection (ATCC, Manassas, VA, USA). Cell proliferation was evaluated in presence of high glucose concentration, so cells was cultured in RPMI 1640 (with high glucose concentration) The medium was supplemented with 10% heat-inactivated FBS, 100 U/mL penicillin, 100 µg/mL streptomycin, and 1% L-glutamine. The cells were grown in a humidified atmosphere of 95% air/5% CO_2_ at 37 °C.

### Cell Viability assessment

H-HepG2 cells were seeded in 96-well plates at the density of 5 × 10^3^ cells/well in complete media in presence of high glucose concentration^10^. The cells were treated with BPA (0.05 µM), Sil (68 µM), and a BPA/Sil combination, for 48 and 72 h.

Cell viability was assessed by adding 3-(4,5-dimethylthiazol-2-yl)-2,5-diphenyl tetrazolium bromide (MTT) solution in culture medium to a final concentration of 5 mg/mL. After a 4 h of incubation at 37 °C, the medium was removed, then the formazan crystals were solubilized by adding 150 μL of dimethyl sulfoxide (DMSO) and by mixing it in an orbital shaker for 5 min. The absorbance at 570 nm was measured by Bio-Rad 550 microplate reader (Bio-Rad Laboratories, Milan, Italy). Cell viability values are expressed as percentage of the control (100%). A control sample contained cells treated with DMSO (final concentration 0.5%) was added to untreated cells. All experiments were performed in triplicate to assess the cause-effect link between the exposition and the results obtained^32^.

### TBARS levels assessment

Samples were incubated with 0.5 mL of 20% acetic acid, pH 3.5, and 0.5 mL of 0.78% aqueous solution of thiobarbituric acid. After heating at 95 °C for 45 minutes, the samples were centrifuged at 4000 r.p.m. for 5 minutes. In supernatant TBARS were quantified by spectrophotometry at 532 nm^19^. Results were expressed as TBARS *µ*M/*µ*g of serum protein. Each data point is the average of triplicate measurements, with each individual experiment performed in duplicate to assess the cause-effect link between the exposition and the results obtained.

### Nitrite levels assessment

NO is rapidly converted into the stable end products nitrite and nitrate. Nitrite was measured by the Griess reaction as reported in literature^33^. Briefly, 100 *µ*L of treated cell colture media was mixed with an equal volume of Griess reagent (0.5% sulfanilamide, 2.5% H_3_PO_4_, and 0.05% naphthylethylene diamine in H_2_O) and incubated for 10 min at room temperature. Absorbance was assayed at 550 nm and compared with a standard curve obtained using sodium nitrite. All The experiments were performed independently to assess the cause-effect link between the exposition and the results obtained.

### Western blot analysis

The effect of BPA, Sil and their combination on the expression of phosphorilized ERK, ERK and Caspase 3 was determined by Western blot analysis. To prepare cell extracts, 2 × 10^6^ HepG2 cells were seeded in tissue culture dishes and incubated with and without BPA, Sil and their combination. The cells were lysed and the proteins were extract as previously described^34^. Equal amounts of cell proteins were resolved on (SDS)-polyacrylamide gels and transferred to nitrocellulose membrane by Trans-blot turbo transfer system (Bio-Rad, Hercules, CA USA). For immunodetection, membranes were incubated overnight at 4 °C with p-ERK, ERK and Caspase 3 antibodies as recommended by the manufacturer. This step was followed by incubation with corresponding horseradish peroxidase (HRP)-conjugated secondary antibody. Protein bands were detected by chemiluminescence detection reagents (Clarity Western ECL Substrate, Biorad). The protein bands were quantified using the ChemiDoc MP system (Biorad). All The experiments were performed independently to assess the cause-effect link between the exposition and the results obtained.

### Extraction of lipids from cell culture medium and mass spectrometry

Lipids were extracted from cell culture medium by a modified methodology of Folch *et al*. (1957). Briefly, for 3 mL of culture medium, methanol (1:4, v/v) was added and mixed rigorously followed by the addition of 3 mL of chloroform and 9 mL of water^35^. The mixture was stirred (5 min) at room temperature and then centrifuged (5 min at 2500 rpm) at 24 °C. Aqueous phase was removed while the lower chloroform phase containing lipids was recovered and concentrated under nitrogen stream to a volume of 0.5 mL. The sample was diluted to 1.0 mL final volume with methanol.

The sample was injected directly, at a flow rate of 10.0 mL min^−1^, into a triple quadrupole spectrometer (API 2000; AB Sciex, Germany) equipped with a TurboIon electrospray source, operating both in the negative and positive modes. The optimized parameters were as follows: capillary temperature 100 °C, IonSprayVoltage: 5.5 kV/−4.5 kV, Declustering Potential: 60 V, Focusing Potential: ±400 V, Entrance Potential: ±10 V. Each analysis required about 1 min. High purity nitrogen was used as the nebulizer and drying gas. Mass spectra were acquired by scanning over the 250–850 m/z range. The Analyst™ software version 1.5.1 (ABI Sciex) was used for instrumentation control and data acquisition. All The experiments were performed independently to assess the cause-effect link between the exposition and the results obtained.

### Extraction of steroids from cell culture medium and mass spectrometry

Steroids extraction from cell culture medium was performed by solid phase extraction (SPE) on molecularly imprinted polymer (MIP) cartridges specific for steroids (AFFINIMIP®SPE POLYNTELL, Polyntell SA, Parigi, Francia).

Cell culture medium (2.5 mL) was added to methanol (1:1, v/v). The mixture was loaded onto AFFINIMIP cartridge, previously conditioned with 3 mL of acetonitrile and 3 mL of ultrapure water. After sample loading, the cartridges were washed with 3 mL of water, 3 mL of water/ acetonitrile (95:5, v/v) and eluted with 3 mL methanol. The eluate was recovered and concentrated under nitrogen stream to a volume of 1 mL.

The solution was injected directly, at a flow rate of 10.0 μL min^−1^, into a triple quadrupole spectrometer (API 2000; AB Sciex, Germany) equipped with a TurboIon electrospray source, operating both in the negative and positive modes. The optimized parameters were as follows: capillary temperature 100 °C, IonSprayVoltage: 5.5 kV/−4.5 kV, Declustering Potential: 90 V, Focusing Potential: ±400 V, Entrance Potential: ±10 V. Each analysis required about 1 min. High purity nitrogen was used as the nebulizer and drying gas. Mass spectra were acquired by scanning over the 200–850 m/z range. The Analyst™ software version 1.5.1 (ABI Sciex) was used for instrumentation control and data acquisition. All The experiments were performed independently to assess the cause-effect link between the exposition and the results obtained.
